# Data Anomaly Detection for Internet of Vehicles Based on Traffic Cellular Automata and Driving Style

**DOI:** 10.3390/s19224926

**Published:** 2019-11-12

**Authors:** Nan Ding, Haoxuan Ma, Chuanguo Zhao, Yanhua Ma, Hongwei Ge

**Affiliations:** School of Computer Science and Technology, Dalian University of Technology, Dalian 116024, China; haoxuan19950916@mail.dlut.edu.cn (H.M.); hwge@dlut.edu.cn (H.G.)

**Keywords:** Internet of Vehicles, data anomaly detection, driving style, Gaussian mixed model, traffic cellular automata

## Abstract

The data validity of safe driving in the Internet of Vehicles (IoV) is the basis of improving the safety of vehicles. Different from a traditional information systems, the data anomaly analysis of vehicle safety driving faces the diversity of data anomaly and the randomness and subjectivity of the driver’s driving behavior. How to combine the characteristics of the IOV data with the driving style analysis to provide effective real-time anomaly data detection has become an important issue in the IOV applications. This paper aims at the critical safety data analysis, considering the large computing cost generated by the real-time anomaly detection of all data in the data package. We preprocess it through the traffic cellular automata model which is built to achieve the ideal abnormal detection effect with limited computing resources. On the basis of this model, the Anomaly Detection based on Driving style (ADD) algorithm is proposed to realize real-time and online detection of anomaly data related to safe driving. Firstly, this paper designs the driving coefficient and proposes a driving style quantization model to represent the driving style of the driver. Then, based on driving style quantization model and vehicle driving state information, a data anomaly detection algorithm is developed by using Gaussian mixture model (GMM). Finally, combining with the application scenarios of multi-vehicle collaboration in the Internet of Vehicles, this paper uses real data sets and simulation data sets to analyze the effectiveness of the proposed ADD algorithm.

## 1. Introduction

The Internet of Vehicles (IOV), a new Ad Hoc Network composed of the basic communication units of mobile vehicles and their surroundings, with the ability of perception, computing, storage and wireless communication running on the road, is an effective measure to improve safe driving. It achieves the communication between vehicle and vehicle, vehicle and road, as well as vehicle and Internet, which is a typical application of Internet of Things (IoT) technology in the field of transportation systems [1]. Multi-vehicle collaboration is a critical technology to solve the problem of unmanned driving, assisted driving and to improve the safety of driving in the IOV. It is the most widely used in IOV application. When dismounted networking is the most widely used field, the most important thing in the scene of assisted driving is to ensure the coordination between vehicles. Data for IOV applications are mainly divided into three categories: critical safety, traffic efficiency, and non-safety. The data of safety driving (e.g., vehicle distance, speed, vehicle control commands), as the basic data of critical safety applications is the foundation of IOV applications. How to guarantee the validity of data in the aspect of safety driving is one of the challenges facing the IOV applications.

Different from the traditional data security prevention methods in the Internet, the data security problems facing the IOV mainly exist in both internal and external causes. On the one hand, the internal safety prevention defects of vehicles are mainly reflected in the existing Internet communication protocols [2], that is, the vehicle lacks an effective verification mechanism for the data transmitted on the Internet, such as Controller Area Network (CAN) protocol. On the other hand, data attacks are diversified due to the open architecture and application of the IOV [3]. For example, hackers remotely controlled the BMW’s onboard system, the Connected Drive, using the security flaw in January 2015. In 2016, Toyota chairman Takeshi Uchiyamada, who has been Chairman of Toyota, claimed that two hackers had also used a computer to access the Prius’ control system before and then removed the car from the driver’s control completely. In June 2018, GCN published a report claiming that connected cars can lie [4], posing a new threat to smart cities.

The research on data anomaly detection method is the key technology for validity and authenticity of interactive data of current multi-vehicle collaboration process in combination with the characteristics of IOV data and driving style analysis. At present, there have been a number of studies on data anomaly detection, such as anomaly detection of traffic flow data based on Turkey smoothing algorithm proposed by Xu et al. [5], and the data cleaning algorithm for redundant data proposed by Wang et al. [6]. However, due to the particularity of IOV application scenario, human behavior is an important parameter that cannot be ignored in the IOV, and driving style has a great influence on the data of IOV. Klauer et al. collected the driving data of 100 incidents; after a deep analysis of the driver’s behavior, they found there was a direct relationship between driving style and vehicle data [7]. Furthermore, different driving styles reflect different characteristics in the data, which is very helpful for us to detect data anomalies. For example, conservative drivers generally have stable data, and frequent data changes are abnormal. For aggressive drivers, this frequent change is a normal behavior. Therefore, this paper combines driving style with anomaly detection. Therefore, this paper aims to propose an anomaly detection algorithm combined with driving style.

Based on the WAVE protocol standard, which defines three types of data for IOV [8]: critical safety, traffic efficiency, and non-safety, this paper aims to analyze security key data, considering that the real-time anomaly detection of all data in the data packet will cause a large computing cost. Thus, in this paper, the traffic cellular automata model is constructed to preprocess the data in order to achieve a desired anomaly detection effect with limited computing resources. On the basis of this model, the Anomaly Detection based on Driving style (ADD) algorithm is proposed based on driving style. In order to quantify driving style better, a new driving style quantization model is proposed, which can more comprehensively quantify driving style via data. The driving style parameter (*e*) obtained from aforementioned model, velocity (*v*), acceleration (*a*), and distance (*d*) are detected via the Gaussian mixture model (GMM). The experiments show that the ADD algorithm proposed in this paper have good performance in data anomaly detection (This work has been published on GitHub. The URL is https://github.com/IoTLabDLUT/Data-Anomaly-Detection-for-Vehicular-Ad Hoc-Networks).

## 2. Relative Works

### 2.1. Traffic Flow Model

According to the definition of traffic flow theory, it is recognized that the measurement scale of traffic flow is divided by two variables [9]: time and space, and the traffic flow model is divided into micro, medium, and macro models. The following model is a typical micro traffic flow model. In the 1950s, John Mitchell and Pipes studied the following theory and built the following theoretical analysis method [10]. After that, scholars did a number of systematic research studies on the following model, published numerous achievements which can be divided into the following categories [11]: the stimulus-response model, the safety distance model, and the physical-mental model. However, the micro-model of a vehicle is also an ideal model with strong assumptions, which is still far from the actual driver’s decision and vehicle behavior (simply describing the local vehicle flow state with less than 10 vehicles on the road, which is mostly used to solve the coordination problem among a small number of vehicles). After the 1990s, researchers built hybrid system models to describe complex traffic flow states based on the theory of a hybrid system and the continuous and discrete characteristics of traffic flow. Lighthill and Whitham first described the one-dimensional theoretical model of fluid dynamics applicable to highway traffic flow on Kinetic waves [12]. After that, many researchers modified the dynamics equation of the model based on traffic practice. Based on the introduction above, Li et al. proposed a new high-order continuous model and carried out a series of calculations to conduct the trend modeling for traffic time series [13].

Prigogine, a famous physicist, and Herman, a famous traffic fluid expert, believed that the influence of individual vehicle behaviors on traffic flow could not be ignored [14]. If using a particle to represent a car, the traffic flow will be considered by many interacting particles gas. Based on the description of the statistical physics in gas motion, the gas dynamic traffic flow model is built by introducing the particle distribution function and establishing a similar Boltzmann equation. Therefore, a number of scholars have proposed many improvements, including changing the relaxation effect term, introducing the correlation between continuous vehicles, investigating the multi-lane effect, and considering imperfect driving, limited space requirements, hybrid vehicles, etc. [15,16]. In addition, many researchers in physics, mathematics, and other disciplines have studied traffic flow problems for the reason of the nonlinearity, complexity, and discreteness of traffic flow. Driven by the science of nonlinearity and complexity, cellular automata model appears, which describes the characteristics of corresponding macro traffic flow by simulating the micro behavior of vehicles. Cellular automata have the characteristics of time discretization, space discretization [17], interaction localization, and dynamic evolution synchronization, which are consistent with the characteristics of traffic flow problems. It has been widely used in the research of traffic flow problems for its simple rules, flexibility, adjustability, and easy simulating [18].

### 2.2. Data Anomaly Detection

Traditional data validity analysis methods focus on simple vehicle data. Lin et al. [19] proposed the pure software CAN bus security mechanism (IDTB-C) to analyze the validity of CAN bus data, which used a CAN message ID table and message counter to generate a message authentication code (MAC) for authentication. Muter et al. [20] designed a method based on sensor detection: multiple sensors with different functions were placed in the corresponding subnet of the CAN bus network, and the data in the CAN bus network was effectively analyzed and detected via the sensors. Xu et al. [5] provided a new way to analyze the validity of traffic flow data by the Turkey smoothing algorithm, and provided an algorithm for recovering invalid data. Their method aimed at screening invalid data. Volkovs et al. [21] introduced a continuous data cleaning framework that can be used in a dynamic data and constraint environment, which allowed both data and their semantics to evolve and suggest repairs based on the accumulated evidence to date. Khodayari et al. [22] took the reaction time as one of the input variables of the model on the basis of the existing models, established the vehicle’s following model by using the neural network to determine whether the data were wrong, and used the traffic data to verify the validity of the model.

In recent years, research also analyzed driving style via data. Precht et al. [23], based on natural driving data from the second strategic highway research program (SHRP 2), found that anger might influence significantly on driving behavior. Klauer et al. [7] collected driving data of 100 accidents, deeply analyzed driver behavior, and then pointed out that there was a direct relationship between driving style and vehicle data. After constructing a determinate relationship between driving style and vehicle data, researchers quantified driving style via models. Qi et al. [24] proposed a new three-layer structure for driving mode by imitating the modified latent Dirichlet allocation (mLDA) model. Langari et al. [25] used the standard deviation value of acceleration to quantify the driving behavior; Murphey et al. [26] used an acceleration derivative as a parameter to analyze driver behavior; Aljaafreh et al. [27] used triaxial accelerations to extract transverse acceleration, longitudinal acceleration, and vertical acceleration as parameters, respectively, which also achieved good results. Other researchers combined multiple data for quantification. Kedar-Dongarkar et al. [28] used acceleration, brake, speed, and throttle to generate a model to analyze driving style. Fugiglando et al. [29] proposed a new method to classify driver behavior by analyzing the selected subset of CAN bus signals, which including accelerator pedal position, brake pedal pressure, steering wheel angle, steering wheel momentum, velocity, revolutions per minute (RPM), and face and lateral acceleration, etc.

In this paper, a traffic cellular automata model that is applicable to variable density is built based on a hybrid system and the cellular automata theory. On the basis of this model, a new driving style quantitative method is proposed combining the acceleration and the vehicle distance. Then, a data quality analysis algorithm, which conducts the real-time data anomaly detection in order to guarantee driving safety, is designed by adding quantized driving style into data anomaly detection.

## 3. Model and Description

### 3.1. Traffic Cellular Automata

Based on the theory of a hybrid system and cellular automata, a traffic flow model of cellular automata adoptive to variable density is constructed. Cellular automata are a dynamical system with discrete space and time, different from a general dynamics model. Instead of being determined by the strict definition of a physical equation or function, it is composed of a series of model construction rule sets, which include the cellular space and states of cellular automata, neighbor states, and space motion rules.

**Figure 1:**
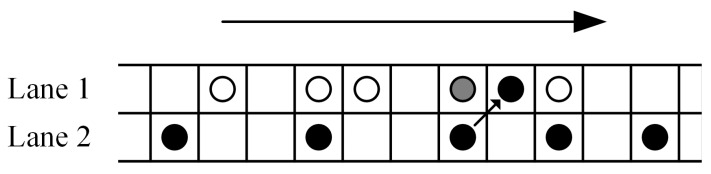
Traffic cellular automata.

In the TCA (Traffic Cellular Automata), as shown in Figure 1, L represents discrete grids of traffic cellular automata model. It is composed of L lanes, each lane has k cellular, and every cellular is dominated by only one vehicle or being idle in a step time. A vehicle can span multiple sequential cellular, the state of the $n_{th}$ vehicle represented by the speed $v_{n}\in \left\{0,1,...,v_{max}\right\}$ and position $x_{n}\in \left\{1,2,...,k\right\}$, and $v_{max}$ represents maximum permitted speed.

The specific process of cellular automata is as follows:

(1) Initialize the traffic cellular automata model

According to previous experience, each cellular automaton corresponds to a road of 7.5 m [30]; however, the TCA model constructed in this way can only be applicable to a specific scenario. For example, on the highway, the vehicle flow density is small, while the vehicle speed and the vehicle distance are large, so that there will be a large number of idle cellular automata in the TCA model, which is not conducive to analysis. Another example is that, in a city with a high traffic density and a small vehicle speed and vehicle distance, all cellular automata will be occupied in the TCA model, or even the same cellular automaton may be occupied by multiple vehicles, so that the TCA model cannot be built correctly. Aiming at this situation, this paper decided to calculate the road length of each cellular automaton according to the average vehicle speed on the road. The formula is as follows:

(1) $$x_{c}=\frac{1}{2}v_{n},$$

where $x_{c}$ is the length of the road that corresponds to each cellular, and $v_{n}$ is the discrete velocity of the *n*th vehicle at this moment.

(2) Calculation of the safe distance

In order to ensure traffic safety, each vehicle must keep a proper distance from the vehicle in front. Too much distance will reduce the road traffic flow, and too small distance is prone to traffic accidents. A safe distance $d_{safe}$ is obtained according to the traffic laws [31], as shown in Table 1.

(3) Update vehicle status based on rule set

The states of all vehicles are updated according to the defined acceleration rules, overtaking/lane-changing rules, deceleration rules, and random slowing rules. The specific evolution rules are given in Section 3.2.

(4) Update the position according to the vehicle status

At the moment of t+1, the position of the vehicle is updated according to the updated speed. When the position is greater than the length of road segment L, the vehicle is regarded as leaving the road segment. Then, remove the vehicle, and the total number of vehicles in the road segment N is reduced. Update the location and remove the vehicle according to the following rules:

(2) $$x_{n}\left(t+1\right)=x_{n}\left(t\right)+v_{n}\left(t+1\right).$$

From the above steps, we can obtain the evolution of our TCA model.

### 3.2. Rule Set of Traffic Cellular Automata

#### 3.2.1. Accelerate Rule

When the distance between the *n*th vehicle and its front vehicle is too large, which this paper stipulates as the safety distance being greater than two times, and the speed does not reach the maximum speed that is $d_{n}\geq 2d_{safe}$ and $v_{n}<v_{\max}$, ($d_{n}$ is the distance between the *n*th vehicle and its front vehicle, $v_{n}$ is the current speed of the *n*th vehicle), and the vehicle can continue to speed up with an acceleration $a_{n}$ (general set to 1). The vehicle accelerates according to the following rules:

(3) $$v_{n}\left(t+1\right)=\min\left(v_{n}\left(t\right)+a_{n},v_{\max}\right).$$

#### 3.2.2. Overtaking/Lane-Changing Rule

When the *n*th vehicle satisfies the overtaking/lane-change condition, it can overtake or change lanes; otherwise, the mandatory deceleration rule is implemented, and the specific conditions are as follows:

(4) $$\left\{\begin{array}{c} d_{n}<\min\left(v_{n}+1,v_{\max}\right),\\ d_{n\text{\_}\mathrm{other}}>d_{n},\\ d_{n\text{\_}back}>d_{safe}, \end{array}\right.$$

where $d_{n\text{\_}other}$ is the distance between the *n*th vehicle and the front vehicle in the adjacent lane (change lane), and $d_{n\text{\_}back}$ is the vehicle distance between the *n*th vehicle and the behind vehicle in the adjacent lane, $d_{safe}$ is the safety distance. The first formula indicates that the distance between the *n*th vehicle and the front vehicle in the same lane is no longer able to satisfy the following accelerating driving conditions at time *t*. The second formula indicates that the driving conditions of adjacent lane are better, which can satisfy the acceleration of the *n*th vehicle. The third formula is the safety condition, indicating that the lane change of the *n*th car will not cause congestion to the behind vehicle in the adjacent lane.

#### 3.2.3. Mandatory Deceleration Rule

When the speed of the *n*th vehicle is too high or the distance from the front vehicle is too small, it is stipulated in this paper that, if $v_{n}>v_{\max}$ or $d_{n}<d_{safe}$, and overtaking/lane change rule is not satisfied, the mandatory deceleration rule should be implemented. The vehicle decelerates according to the following rule:

(5) $$v_{n}\left(t+1\right)=\max\left(v_{n}\left(t\right)-a_{n},0\right).$$

#### 3.2.4. Random Slowing Rule

In order to get closer to the measured data, according to the sensitive driving model proposed by Larraga et al. [30], considering the driver’s uncertain behavior, the vehicle speed is randomly slowed, and the random slowing probability $p_{n}$ is introduced to randomly slow with acceleration $a_{n}$. The vehicle is randomly slowed according to the following rules:

(6) $$v_{n}\left(t+1\right)=\left\{\begin{array}{cc} \max\left(v_{n}\left(t\right)-a_{n},0\right), & p_{n}\leq \frac{1}{3},\\ v_{n}\left(t\right), & p_{n}>\frac{1}{3}. \end{array}\right.$$

The rule is to simulate random behavior, and $p_{n}$ is a random value.

### 3.3. Driving Style Quantization Model

**Figure 2:**
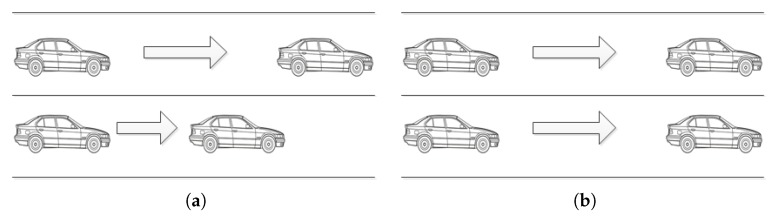
The defect of unilateral quantization.

According to the multidimensional driving style table, driving styles are classified in a broad sense [32]. This paper divides driving styles into three types: cautious type (C), normal type (N), and aggressive type (A). There are generally two angles for quantifying driving style via data. The first Angle is quantized according to the speed and acceleration of vehicles. The driver style recognition coefficient $R_{driver}$ proposed by Murphey et al. [26] is a very classical algorithm. The second Angle is quantized according to the vehicle distance. At present, all of the research is simply quantized from one aspect, so that there is a certain deviation from the actual situation, which will lead to inaccurate quantization results. As shown in Figure 2: (a) When the distance between the vehicle and its front car is too large, the behind car can be considered as free driving, and frequent changes in acceleration are normal. However, from the perspective of acceleration, the driver of the behind vehicle will be judged as aggressive with a high probability; (b) In the case of the same vehicle distance, drivers with frequent changes in acceleration (speed) must have different driving style types from drivers with stable acceleration (speed), but it cannot be reflected by vehicle distance analysis alone.

In this paper, based on the driver style recognition coefficient $R_{driver}$ proposed by Murphey et al. [26], a new concept–driving style recognition coefficient $R_{de}$ is proposed by combining acceleration and vehicle distance, and driving style is quantified by using acceleration and vehicle distance.

Define vehicle distance ratio *p*:

(7) $$p=\frac{s}{d},$$

where *d* is the vehicle distance, and *s* is the shortest safe distance, which is obtained according to the safe distance specified in the traffic laws [31], as shown in Table 1.

If the vehicle distance *d* is less than the safe distance *s*, the driver is considered to be aggressive (A), and the driving style recognition coefficient $R_{de}$ is not required to be calculated.

Then, the driver style recognition coefficient $R_{driver}$ is calculated as follows:

(8) $$J_{\left(t\right)}=\frac{d^{2}v\left(t\right)}{dt^{2}},$$

(9) $$R_{\;\mathrm{driver}\;}=\frac{R_{J}}{\bar{J}}.$$

*J${}_{\left(t\right)}$* is the impact, defined in physics as the rate of acceleration change; $\bar{J}$ is the average impact of normal drivers under the same condition; *R${}_{J}$* is the standard deviation of the impact within a time period with a time window ω. Through a large number of experiments and studies, Murphey suggested setting the time window as 6 s or 9 s, which will have a high recognition precision [26]. This paper refers to this setting.

Driving style recognition coefficient $R_{de}$ can be expressed as:

(10) $$R_{de}=R_{\;\mathrm{driver}\;}\times p=\frac{R_{J}}{\bar{J}}\times \frac{s}{d}.$$

Finally, the calculated driving style coefficient is compared with two thresholds: the normal threshold *Norm${}_{threshold}$* and the aggressive threshold *Agg${}_{threshold}$*. Through a lot of experiments, this paper suggests that the two thresholds are 0.5 and 1.0, respectively.

## 4. Add Algorithm: Anomaly Detection Based on Driving Style

Considering that part of the attacking data in the actual situation will not affect the safety of driving, regarding it as normal data can effectively reduce the calculation cost without affecting the accuracy, thus this paper adopts the following methods to preprocess all the data:

(11) $$C=\left\{\begin{array}{cc} 0, & \exists f\in F\to \;a=f(d),\\ 1, & \;\mathrm{else}\;. \end{array}\right.$$

*C* is the preliminary screening index for anomaly, and *C* is 1 for possible anomaly, while *C* is 0 for normal data. *F* is the rule set of cellular automata traffic flow model, *f* is one of these rules, *a* is the decision of the vehicle, and *d* is the data of the vehicle. Equation (11) shows that, if the vehicle data can keep the TCA system steady, we believe that the data are normal data; otherwise, it is considered suspected abnormal data and proceeds to the next step for further judgment.

The formula for the driving style recognition coefficient $R_{de}$ is given in Section 3.3.

After obtaining $R_{de}$, this paper converted it into the corresponding driving style coefficient *e*, in order to conduct anomaly detection via GMM later. The corresponding relation of $R_{de}$ and *e* is shown as Table 2:

Where *Norm${}_{threshold}$* is normal threshold, *Agg${}_{threshold}$* is aggressive threshold (according to the experiment results, it is suggested that two thresholds are valued 0.5 and 1.5 respectively).

### 4.1. Gaussian Mixed Model (GMM)

After obtaining driving style parameter *e*, data anomaly detection is conducted. For the sake of research, this paper extracts the velocity *v*, acceleration *a*, distance *d*, and quantized driving style parameter *e* from the data packet for anomaly detection.

**Figure 3:**
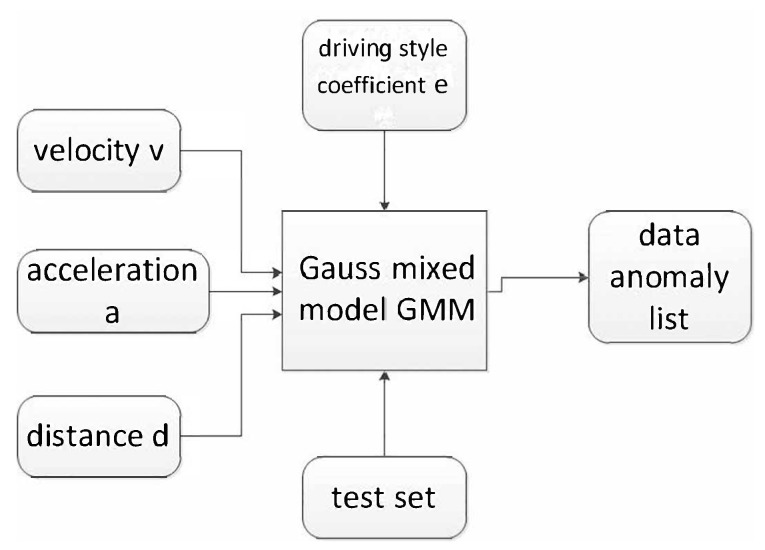
Gaussian Mixed Model parameter.

Since Gaussian mixture model (GMM) is applicable to continuous variables and can reflect the correlation between dimensions, this paper uses the GMM to carry out this work, as shown in Figure 3:

GMM is often used in clustering. Taking a point in GMM distribution randomly can be divided into two steps: first, choose one component from *K* components randomly; the probability of selecting each component is actually its coefficient $\pi_{k}$. After selecting components, separately consider selecting one point from this component’s distribution; here, it has returned to normal Gaussian distribution and has converted to known issues. Thus, GMM is used for clustering, and we only need to deduce the probability distribution of GMM according to the data.

The random variable *X* is set; then, the Gaussian mixture model can be expressed as follows:

(12) $$P\left(X\right)=\sum_{k=1}^{K}\pi_{k}N\left(x|\mu_{k},\Sigma k\right),$$

where $N(x|\mu_{k},\Sigma K)$ is the kth component of the mixture model. Given two clusters, which can be represented by two two-dimensional Gaussian distributions, then the component *K* = 2. $\pi_{k}$ is the mixture coefficient that satisfies:

(13) $$\sum_{k}^{K}\pi_{k}=1,0\leq \pi_{k}\leq 1,$$

where $\pi_{k}$ is equivalent to the weight of each component $N(x|\mu_{k},\Sigma K)$; then, the form of two clustering GMM is shown in the formula:

(14) $$P\left(X\right)=\pi_{1}N\left(x|\mu_{1},\Sigma 1\right)+\pi_{2}N\left(x|\mu_{2},\Sigma 2\right).$$

The problem of GMM parameter estimation is how to determine the value of $\pi_{1}$ and $\pi_{2}$ automatically based on data. To solve this problem, we can use the Expect–Maximization (EM) algorithm. With the EM algorithm, we can iteratively calculate (*$\pi_{k}$, $x_{k}$, Σk*) in GMM.

The EM algorithm has two steps. The first step is to obtain the rough value of the estimating parameter; the second step is to use the value of the first step to maximize likelihood function. Thus, the likelihood function of GMM should be obtained first; there are three parameters in GMM model to estimate: *π, μ* and Σ. Rewrite Equation (12):

(15) $$P\left(x|\pi ,\mu ,\Sigma \right)=\sum_{k-1}^{K}\pi_{k}N\left(x|\mu_{k},\Sigma k\right).$$

In order to estimate the three parameters, the maximum likelihood function of the three parameters needs to be solved respectively. First, solve the maximum likelihood function of $\mu_{k}$. Take the logarithm of Formula (15) and then take the derivative of $\mu_{k}$ and set the derivative to *0*, the maximum likelihood function can be obtained:

(16) $$0=-\sum_{n-1}^{N}\frac{\pi_{k}N\left(x_{n}|\mu_{k},\Sigma k\right)}{\sum j\pi_{j}N\left(x_{n}|\mu_{j},\Sigma j\right)}\sum k\left(x_{n}-\mu_{k}\right).$$

Both sides multiply $\sum k^{-1}$, rearranging to get:

(17) $$\mu_{k}=\frac{1}{N_{k}}\sum_{n-1}^{N}\gamma \left(z_{nk}\right)x_{n},$$

where:

(18) $$N_{k}=\sum_{n-1}^{N}\gamma \left(Z_{nk}\right),$$

(19) $$\gamma \left(z_{k}\right)=p\left(z_{k}=1|x\right)=\frac{\pi_{k}N\left(x|\mu_{k},\Sigma k\right)}{\sum_{j-1}^{K}\pi_{j}N\left(x|\mu_{j},\Sigma j\right).}$$

As shown in Formula (19), $\gamma (z_{k})$ is defined to represent the posterior probability of the $k_{th}$ component. In Formulas (17) and (18), *N* is the number of points, then $\gamma (z_{nk})$ can represent the posterior probability of *n* ($x_{n}$) belonging to cluster *k*, $\mu_{k}$ is the weighted average of all points, and $\sum_{n-1}^{N}\gamma \left(z_{nk}\right)$ is the weight of each point, which is related to the $k_{th}$ cluster.

Calculate the maximum likelihood function of Σk in the same way; we can obtain:

(20) $$\Sigma k=\frac{1}{N_{k}}\sum_{n-1}^{N}\gamma \left(z_{nk}\right)\left(x_{n}-\mu_{k}\right){\left(x_{n}-\mu_{k}\right)}^{T}.$$

Finally, the maximum likelihood function of $\pi_{k}$ remains; it can be regarded as the prior probability of $z_{k}=1$. Note that there are constraints $\sum_{k-1}^{K}\pi_{k}=1$ of $\pi_{k}$, so we need to add the Lagrangian operator:

(21) $$\ln p\left(x|\pi ,\mu ,\Sigma \right)+\lambda \left(\sum_{k-1}^{K}\pi_{k}-1\right).$$

To calculate the maximum likelihood function of $\pi_{k}$ above in the same way, we can obtain:

(22) $$0=\sum_{n-1}^{N}\frac{N\left(x_{n}|\mu_{k},\Sigma k\right)}{\sum j\pi_{j}N\left(x_{n}|\mu_{j},\Sigma j\right)}+\lambda .$$

Both sides multiply $\pi_{k}$, and we can obtain λ=−N, which leads to a more concise expression of $\pi_{k}$:

(23) $$\pi_{k}=\frac{N_{k}}{N}.$$

Using the EM algorithm to estimate GMM parameters is to maximize Formulas (17), (20) and (23), Formulas (17), (19), (20) and (23) are required. First, assign the initial value of *π*, *μ* and Σ, which is substituted into Formula (19) to obtain $\gamma (z_{nk})$. Then, substitute $\gamma (z_{nk})$ into Formulas (18), (20) and (23) to obtain *$\pi_{k}$*, *$\mu_{k}$* and Σk. Subsequently, substitute $\pi_{k}$, $\mu_{k}$ and Σk into Formula (19) to obtain new $\gamma (z_{nk})$, then substitute new $\gamma (z_{nk})$ into Formulas (17), (20) and (23). Repeat the former steps until the algorithm converges.

### 4.2. Add Algorithm

**Figure 4:**
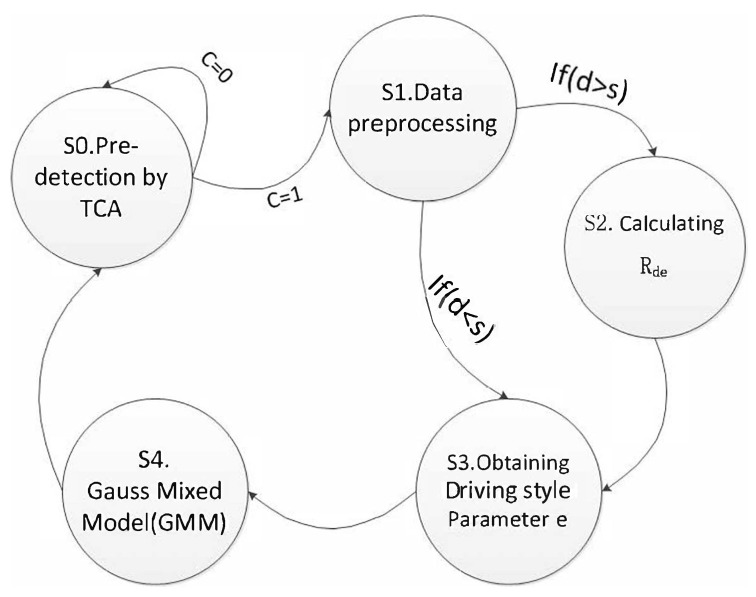
State transition diagram.

Anomaly detection algorithm of IOV data, ADD, is proposed based on driving style. The state transition diagram of ADD algorithm is shown in Figure 4.

The main process is as follows:

S0: Pre-detection by traffic flow model. If the vehicle data conforms to the TCA model (*C* = 0), determine it as normal data and continue S0; if the vehicle data does not conform to the TCA model (*C* = 1), anomalies may exist and further comprehensive determination is needed, and proceed to S1.

S1: Data preprocessing. Speed *v*, acceleration *a* and distance *d* are extracted from the data packet. The distance *d* and the minimum safe distance *s* are compared. If d≥s, proceed to S2; otherwise, proceed to S3.

S2: The driving style recognition coefficient $R_{de}$ is calculated according to the Formula (10).

S3: The driving style coefficient *e* is obtained according to the comparison table of driving style parameters.

S4: The obtained driving style coefficient *e*, velocity *v*, acceleration *a*, and distance *d* are used to obtain the anomalies list via the Gaussian mixture model (GMM).For a given m-dimensional data set $\{x^{1},x^{2},...,x^{m}\},x\in R$, using a Gaussian mixture model to calculate mathematical expectation μ and build the covariance matrix Σ of all the characteristics, as shown in Formulas (24) and (25):

(24) $$\mu =\frac{1}{m}\sum_{i=1}^{m}x^{i},$$

(25) $$\Sigma =\frac{1}{m}\sum_{i=1}^{m}\left(x^{i}-\mu \right){\left(x^{i}-\mu \right)}^{T}.$$

The probability density function is as shown in Formula (26):

(26) $$P\left(x;\mu ,\sigma^{2}\right)=\frac{1}{{\left(2\pi \right)}^{\frac{\pi }{2}}{\left|\Sigma \right|}^{\frac{1}{2}}}exp\left(-\frac{1}{2}{\left(x-\mu \right)}^{T}\Sigma^{-1}\left(x-\mu \right)\right).$$

The probability density function calculated in Formula (26) is used to judge the new data, and $P_{\left(x\right)}$ can be compared with the adaptive threshold to detect anomaly data; finally, the anomalies list can be obtained through output.

## 5. Experiment and Analysis

In this paper, the ADD algorithm is proposed to analyze the validity of IOV data. Two data sets are used in the simulation experiment.

Data set 1: NGSIM data set for experimental simulation [33]. Researchers collected detailed vehicle trajectory data on southbound US 101 and Lankershim Boulevard in Los Angeles, CA, USA eastbound I-80 in Emeryville, CA, USA and Peachtree Street in Atlanta, GA, USA. Data were collected through a network of synchronized digital video cameras. NGVIDEO, a customized software application developed for the NGSIM program (Dataset 1.1; USDOT; Los Angeles, California; Emeryville, California; Atlanta, Georgia; America), transcribed the vehicle trajectory data from the video. This vehicle trajectory data provided the precise location of each vehicle within the study area every 0.1 s, resulting in detailed lane positions and locations relative to other vehicles.

**Figure 5:**
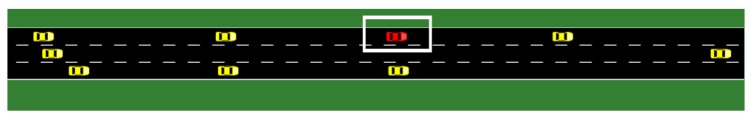
Simulation of Urban MObility simulation platform.

Data set 2: The self-made data set simulated by Simulation of Urban MObility (SUMO), using a “five-car model” and custom vehicle to generate autonomous vehicles required by the experiment. In order to distinguish the two different types of vehicles, we use red vehicles to represent autonomous vehicles and yellow vehicles to represent environmental vehicles, as shown in Figure 5. Then, collect the simulated data and analyze it as the interactive data between vehicles.

**Figure 6:**
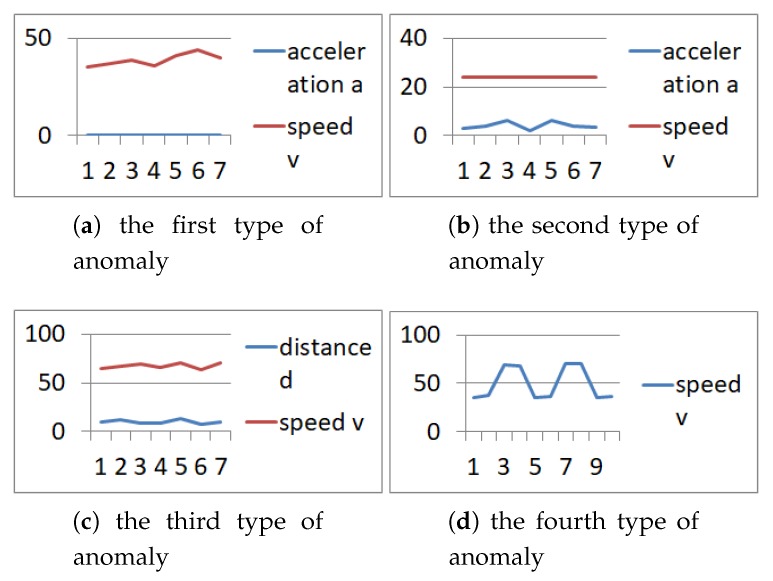
Data anomalies definition.

This paper only changes the speed to simulate anomaly data. According to the actual situation and the investigation of Chen et al. [34], four types of anomaly data are defined, as Figure 6:

1. a=0,|Δv|≥5%, the acceleration is 0, but the speed changes;

2. a≠0,|Δv|≤5%, the acceleration is not 0, but the speed remains unchanged;

3. The distance(*d*) is too small when the speed or acceleration is large;

4. |Δv|>50%, a step occurs in speed or acceleration.

In order to prove the performance of the algorithm proposed in this paper, two algorithms are adopted for comparison: (1) HTM algorithm: Hole et al. [35] carried out anomaly detection on the time series data, whose data set was derived from the collected time series data of various industries, including the vehicle speed in the IOV. In this paper, the HTM algorithm is only used for anomaly detection of one-dimensional data, velocity v, for comparison. (2) LSTM algorithm: Filonov et al. [36] adopted a method based on LSTM neural network to monitor and detect anomalies in multivariable time series data. In this paper, the LSTM algorithm is used to detect the anomalies of the three-dimensional IOV data (speed, acceleration, distance) without driving style for comparison.

**Figure 7:**
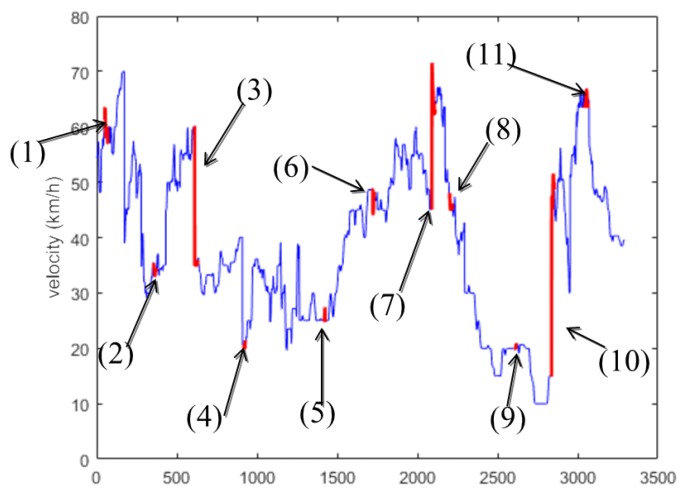
Speed diagram of training set.

In this paper, ten cars in the NGSIM data set are randomly selected as the GMM training data set. Figure 7 shows the speed trend of the training set, where the velocity step between two vehicles is marked as anomaly 4.

There are 11 anomaly intervals in the graph above, the specific situation is as follows:

Anomaly intervals (5) and (8): when the acceleration is 0, the velocity changes (anomaly 1);

Anomaly intervals (6) and (9): when the acceleration is not 0, the velocity remains unchanged (anomaly 2);

Anomaly interval (1) and (11): when the vehicle distance d is small, the speed or acceleration is large (anomaly 3);

Anomaly intervals (2), (3), (4), (7), and (10): when velocity or acceleration step is generated, which is not normalcy (anomaly 4).

**Figure 8:**
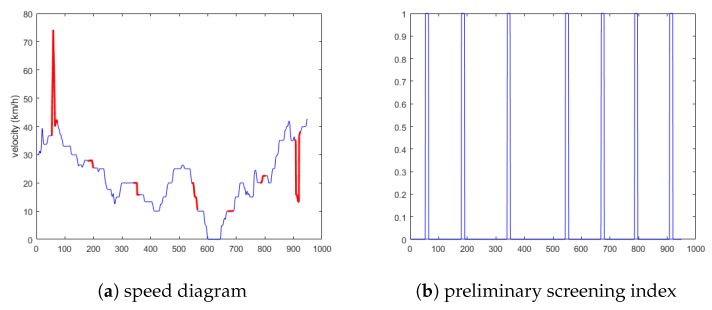
Schematic diagram of the first situation.

GMM test data set is composed of two parts. Test set 1 is the vehicles randomly selected from the NGSIM data set, and the following three test sets are obtained:

State 1 selects the vehicles as 14, 233, 999, and 2333. The anomaly data include all four anomaly conditions. The schematic diagram is shown in Figure 8a. Figure 8b is the preliminary screening index of anomalies obtained via the cellular automata traffic flow model.

**Figure 9:**
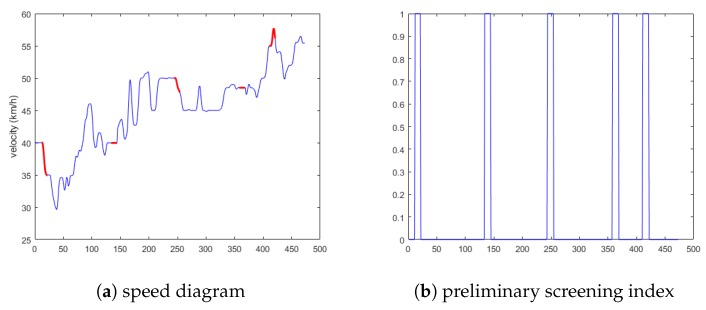
Schematic diagram of the second situation.

State 2 selects the vehicles as 28 and 78, and the anomaly data includes anomaly 1, 2, and 3. All these three anomalies are considered to be related to each other; the schematic diagram is shown in Figure 9a. Figure 9b is the preliminary screening index of anomalies obtained via the cellular automata traffic flow model.

**Figure 10:**
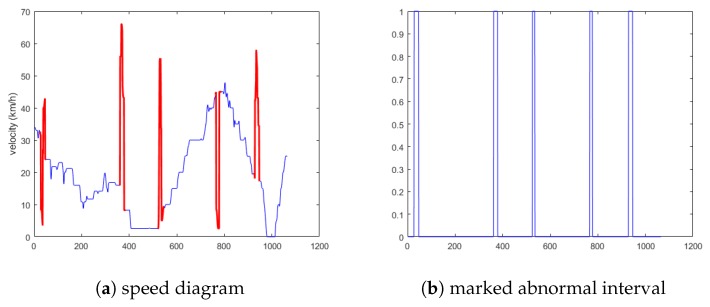
Schematic diagram of the third situation.

State 3 selects the vehicles as 59 and 1202, and there is only anomaly 4. In this case, there was only step anomaly of speed, as shown in Figure 10a. Figure 10b is the preliminary screening index of anomalies obtained via the cellular automata traffic flow model.

**Figure 11:**
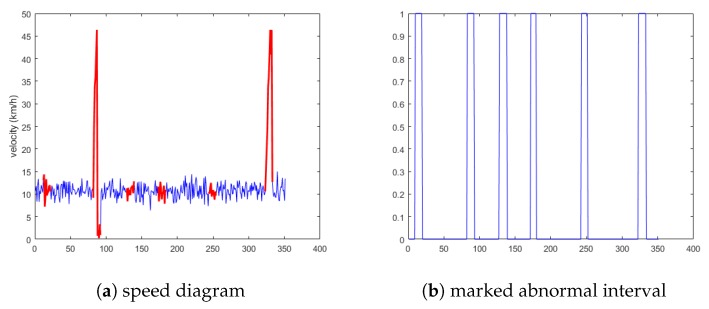
Schematic diagram of SUMO data set.

Test set 2 is obtained by adding the anomaly data (including all four anomalies, in the first situation) into the SUMO simulation data set, as shown in Figure 11.

### 5.1. Experimental Results and Analysis

**Figure 12:**
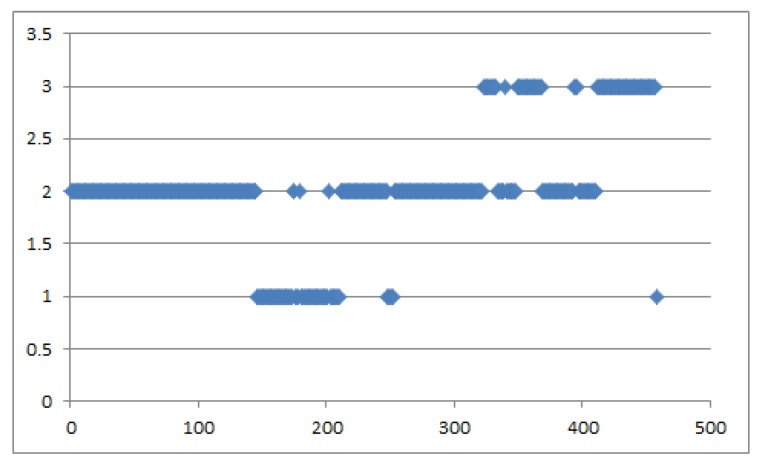
The driving style quantification result.

According to the actual situation and the study of Murphey et al. [26], the changes of driving style are not a mutation process reflected in the data; thus, this paper proposes that the driving style *e* is a transient data, which is quantified by a sliding time window (it is suggested that the time window should be consistent with ω). The driving style quantification result of a vehicle (Id: 562) is shown in Figure 12.

When comparing the experimental results of the test set, the three parameters–Precision, Recall and $F_{1}$ score, which are commonly used in the field of data anomaly identification, are selected as the evaluation criteria. The common method for calculating Precision, Recall, and $F_{1}$ scores is shown in Formulas (27) to (29):

(27) $$Precision=\frac{T_{p}}{T_{p}+F_{p}},$$

(28) $$Recall=\frac{T_{p}}{T_{p}+F_{n}},$$

(29) $$F_{1}=\frac{2*Precision*Recall}{Recall+Precision},$$

where $T_{p}$ represents the number of correctly detected, $F_{p}$ represents the number of false positives, and $F_{n}$ represents the number of false negatives. As shown in Formulas (27)–(29), the number of $T_{p}$, $F_{p}$ and $F_{n}$ will determine the Precision and Recall. The precision is used for judging how sensitive the anomaly detection algorithm is to anomalies. The recall rate reflects the ability of the algorithm to detect anomalies. The accuracy and recall values together affect the final $F_{1}$ score, which represents the overall performance of the anomaly detection algorithm.

#### 5.1.1. Experimental Results Analysis of the First Situation

In the first situation, data anomaly detection results are shown in Table 3 and Table 4, where Table 3 is the result of test set 1 (NGSIM data set) and Table 4 is the result of test set 2 (SUMO simulation data set).

It can be seen from the table that the HTM algorithm only performs anomaly detection on one-dimensional speed data, and its Precision is generally high, but Recall is very low because the HTM algorithm can detect step anomalies well and has low false positive ($F_{p}$). The correlation between multidimensional data cannot be taken into account in the HTM algorithm, which will result in high false negative ($F_{n}$). Therefore, the Recall of each group is very low, and the final calculated $F_{1}$ score is also very low.

The Precision of the LSTM algorithm is not much different from that of the ADD algorithm; however, the Recall of the LSTM algorithm is significantly higher than that of the former. Because the ADD algorithm takes into account the driving style, the detection is more comprehensive, the false negative ($F_{n}$) detection result is very low, and the final calculated $F_{1}$ score is also significantly higher than the other two algorithms.

#### 5.1.2. Experimental Results Analysis of the Second Situation

In the second situation, the results of anomaly data detection are shown in Table 5. The HTM algorithm used for detection has low Precision and Recall, and the final calculated $F_{1}$ score will be far lower than the latter two. The results of LSTM algorithm and ADD algorithm are not much different from the first situation. The Precision of the ADD algorithm is similar to the former, but the Recall is higher than the former, and the final $F_{1}$ score is also higher than the former.

#### 5.1.3. Experimental Results Analysis of the Third Situation

In the third situation, the results of anomaly data detection are shown in Table 6. There is only velocity step anomaly, and any algorithm used for detection will have high Precision and Recall. The final calculated $F_{1}$ score will be close to each other. The detection effects of the three algorithms will not be much different.

We can clearly determine that the performance used multidimensional data to carry out validity analysis is much better than using only one-dimensional speed data via the comparison among three situations because it will not consider the correlation between multidimensional data, which will lead to high false negative results and a very low final calculated $F_{1}$ score. In the same situations above, the method of adding the driving style parameter *e* is better than the method irrespective of that. Although it will create unnecessary mistakes and increase false positive ($F_{p}$), more comprehensive calculation can detect more anomalies, decrease false negative ($F_{n}$), and improve Recall; finally, the $F_{1}$ score calculated by combining Precision and Recall will be significantly higher than the method irrespective of driving style.

## 6. Conclusions

This paper builds a traffic cellular automata model based on the hybrid system and cellular automata theory, and preprocesses the data of IOV according to this model, in order to achieve an ideal anomaly detection effect with limited computing resources. On the basis of this model, a new driving style quantization method is proposed by combining acceleration and vehicle distance, and the ADD algorithm, a data validity detection method for IOV, is proposed based on driving style demonstrated in Section 4, which is more reasonable. Then, NGSIM data sets and SUMO simulation data are used to verify the proposed method. Experiments show that adding driving style parameters to data validity analysis will bring better performance, and the method proposed in this paper is reasonable and feasible.

## Figures and Tables

**Table 1:** Safety distance.

Driving Type	Condition (km/h)	Safety Distance
high speed driving	v>100	s≥100 m
fast speed driving	70<v≤100	s≥80 m
medium speed driving	40<v≤70	s≥60 m
low speed driving	20<v≤40	s≥30 m
turtle speed driving	v≤20	s≥10 m

**Table 2:** Driving style coefficient contrast table.

$R_{\mathrm{de}}$	Driving Style	Coefficient e
$R_{de}<Norm_{threshold}$	Cautious (C)	1
$Norm\leq R_{de}\leq Agg$	Normal (N)	2
$R_{de}>Agg_{threshold}$	Aggressive (A)	3

**Table 3:** Results of test set 1.

(a) Precision			
ID	HTM	LSTM	ADD
14	0.76	0.89	0.90
233	0.67	0.86	0.90
999	0.60	0.81	0.88
2333	0.74	0.89	0.91
AVG	0.69	0.86	0.90
(b) Recall			
ID	HTM	LSTM	ADD
14	0.36	0.81	0.94
233	0.39	0.87	0.95
999	0.43	0.89	0.86
2333	0.44	0.95	0.95
AVG	0.40	0.88	0.95
(c) F1 score			
ID	HTM	LSTM	ADD
14	0.49	0.84	0.92
233	0.49	0.86	0.92
999	0.50	0.85	0.92
2333	0.55	0.92	0.93
AVG	0.51	0.87	0.92

HTM: Hierarchical Temporal Memory. LSTM: Long short-term memory. ADD: Anomaly Detection Based on Driving Style.

**Table 4:** Results of test set 2.

	pre	rec	f1
HTM	0.75	0.36	0.49
GMM	0.79	0.64	0.71
ADD	0.83	0.74	0.78

HTM: Hierarchical Temporal Memory. LSTM: Long short-term memory. ADD: Anomaly Detection Based on Driving Style. pre: Precision; rec: Recall; f1: F1 score.

**Table 5:** Results of the second situation.

(a) Precision			
ID	HTM	LSTM	ADD
28	0.31	0.77	0.84
78	0.30	0.81	0.82
AVG	0.30	0.79	0.83
(b) Recall			
ID	HTM	LSTM	ADD
28	0.16	0.67	0.87
78	0.13	0.72	0.91
AVG	0.14	0.69	0.89
(c) F1 score			
ID	HTM	LSTM	ADD
28	0.21	0.72	0.86
78	0.18	0.76	0.86
AVG	0.19	0.74	0.86

HTM: Hierarchical Temporal Memory. LSTM: Long short-term memory. ADD: Anomaly Detection Based on Driving Style.

**Table 6:** Results of the third situation.

(a) Precision			
ID	HTM	LSTM	ADD
59	0.95	0.89	0.91
1202	0.91	0.82	0.86
AVG	0.93	0.86	0.88
(b) Recall			
ID	HTM	LSTM	ADD
59	0.92	0.95	0.95
1202	0.91	0.92	0.94
AVG	0.91	0.93	0.94
(c) F1 score			
ID	HTM	LSTM	ADD
59	0.93	0.92	0.93
1202	0.91	0.87	0.89
AVG	0.91	0.89	0.91

HTM: Hierarchical Temporal Memory. LSTM: Long short-term memory. ADD: Anomaly Detection Based on Driving Style.
